# Clinical features of the first critical case of acute encephalitis caused by the avian influenza A (H5N6) virus

**DOI:** 10.1080/22221751.2022.2122584

**Published:** 2022-10-26

**Authors:** Libing Zhang, Kaituo Liu, Qin Su, Xiao Chen, Xiaoquan Wang, Qingfeng Li, Wenlei Wang, Xuhua Mao, Jinmei Xu, Xin Zhou, Qin Xu, Le Zhou, Xiufan Liu, Pinghu Zhang

**Affiliations:** a Department of Pediatrics of the Affiliated Hospital of Yangzhou University, Yangzhou University, Yangzhou, People’s Republic of China; b Joint International Research Laboratory of Agriculture and Agri-Product Safety, The Ministry of Education of China, Yangzhou University, Yangzhou, People’s Republic of China; c Jiangsu Key Laboratory of Integrated Traditional Chinese and Western Medicine for Prevention and Treatment of Senile Diseases, Institute of Translational Medicine, Medical College of Yangzhou University, Yangzhou, People’s Republic of China; d Animal Infectious Disease Laboratory, College of Veterinary Medicine, Yangzhou University, Yangzhou, People's Republic of China; e Department of Clinical Laboratory, The Affiliated Yixing Clinical School of Medical School of Yangzhou University, Yangzhou, People’s Republic of China; f Yangzhou Center for Disease Control and Prevention, Yangzhou, People’s Republic of China; g Jiangsu Key Laboratory of Zoonosis, Yangzhou University, Yangzhou, People’s Republic of China.

**Keywords:** Acute encephalitis, H5N6, influenza A virus, wild waterfowls, phylogenetic analysis

## Abstract

Highly pathogenic avian influenza viruses (HPAIV), such as H5N1, H5N6, and H7N9, have been reported to frequently infect humans, but acute encephalitis caused by HPAIV in humans has been rarely reported. We report the first critical case of acute encephalitis with mild pneumonia caused by the H5N6 virus. On January 25 of 2022, a 6-year-old girl with severe neurological symptoms was admitted to our hospital and rapidly developed into seizures and coma. Brain imaging showed abnormalities. Electroencephalogram (EEG) presented abnormal slow waves. Cerebrospinal fluid (CSF) contained elevated protein (1.64 g/L) and white cells (546 × 10^6^/L). Laboratory investigations revealed abnormally elevated transaminases, lactate dehydrogenase, and cytokines in serum. A novel reassortant H5N6 virus was identified from the patient’s serum, CSF, and tracheal aspirate specimens. Phylogenic analysis indicated that this virus was a novel reassortant avian-origin influenza A (H5N6) virus that belonged to clade 2.3.4.4b. This patient was diagnosed with acute encephalitis and discharged from the hospital accompanied by a language barrier. An epidemiological investigation confirmed that wild waterfowls were the direct source of infection in this case. Our study highlights the urgent need to pay attention to acute encephalitis caused by HPAIV.

## Introduction

Due to the high lethality in humans, highly pathogenic avian influenza viruses (HPAIV), such as H5N1, H7N9, and H5N6, have posed a great threat to human health [1–3]. During the past 20 years, the H5N1 virus has caused 863 cases including 455 deaths in the world [4]. However, since 2014 the H5N6 reassortant virus has been circulating in poultry and occasionally infected humans in China [3,5]. To date, 78 cases (32 deaths) of the H5N6 virus have been reported, of which 77 cases occurred in China [6]. Notably, most cases of H5N6 infection occurred in 2021 and 2022 [5–8]. Different from seasonal influenza, most patients infected with HPAIV presented primarily with severe respiratory symptoms, and neurological symptoms associated with acute encephalitis in humans are still extremely rare. Here, we report the first critical case of acute encephalitis diagnosed by identifying the H5N6 virus from cerebrospinal fluid (CSF), throat, and serum specimens in a 6-year-old girl who presented with seizures and coma. The clinical features of the patient with acute encephalitis are described and the epidemiological investigation was established. She was discharged from the hospital, but brain imaging and electroencephalogram (EEG) still indicated abnormalities. All evidence indicated the most likely resource of infection might be the wild waterfowl. Our study highlights that attention needs to be paid to viral encephalitis caused by HPAIV (H5N6) transmitted by migratory birds.

## Materials and methods

### Clinical data collection

All clinical data about the patient were collected from illness onset to discharge including clinical signs and symptoms, chest radiography, chest computed tomography, brain computed tomography, magnetic resonance imaging (MRI), EEG, clinical laboratory testing, antiviral or antibacterial treatment, symptomatic treatment, supportive care, and intensive care. After the diagnosis has been identified, the patient’s village and house were surveyed. Her family and other close contacts with potential exposure risks were identified and interviewed. An epidemiological investigation and a risk assessment of these close contacts were evaluated. Written informed consent from the patient was obtained. The ethics committee of the affiliated hospital of Yangzhou university approved this study. The diagnosis for virus isolation was performed in the biosafety level 3 (BSL3) or the animal biosafety level 3 facility.

### Pathogenic investigation

Blood, urine, CSF, sputum, and tracheal aspirate specimens were collected on day 6 of the illness onset for pathogenic bacterial, fungal, and viral isolation and culture. Sera and CSF were collected on day 7 of illness onset for metagenomic next-generation sequencing (mNGS) to identify the potential pathogens [9]. Serum specimens were collected on day 7 of illness onset and used to examine HBV antigen, HCV antigen, HIV antigen, HIV antibodies, influenza A virus H1 and H3 subtype antibodies (IgM), influenza B virus antibodies (IgM), Adenovirus (ADV) antibodies (IgM), respiratory syncytia (RSV) antibodies (IgM), *chlamydia pneumonia* antibodies (IgM), *mycoplasma pneumonia* antibodies (IgM and IgG), human parainfluenza virus antigen and antibodies (IgM), and EBV antibodies (IgM, IgG, and IgA) with ELISA. MDCK cells and 10-old-day specific pathogen-free (SPF) embryonated chicken eggs were used to isolate the virus, as previously described [10]. The virus titres were examined with a haemagglutination test using chicken red blood cells. Serum and CSF antibody titres against the H5N6 virus were measured by haemagglutination inhibition (HI) assay according to the standard protocols of OIE [11].

RNA from the serum, CSF, and tracheal aspirate specimens was extracted with an RNA isolator (Vazyme, China), according to the manufacturer’s instructions. Extracted RNA was reverse-transcribed to cDNA with HiScript II 1st Strand cDNA Synthesis Kit (Vazyme, China). Specific real-time PCR assays for influenza A viruses, influenza B viruses, enteroviruses, parainfluenza viruses, respiratory adenoviruses, human metapneumoviruses, respiratory syncytial viruses, human rhinoviruses, human bocaviruses, SARS-CoV-2, *mycoplasma pneumoniae*, *pneumonia chlamydia*, *legionella pneumophila*, *pseudomonas aeruginosa*, *streptococcus pneumoniae*, *klebsiella pneumoniae*, *group A streptococcus*, *haemophilus influenzae*, and *staphylococcus aureus* were performed to identify the potential pathogen. The primers are available on request. To clarify the potential transmission routes of this case, epidemiological surveillance for faecal samples collected from local wild waterfowls was performed by isolating the virus with 10-day-old SPF embryonated chicken eggs, as previously described [12].

DNA from the patient’s CSF and sera for mNGS was extracted using a QIAamp® UCP Pathogen DNA Kit (Qiagen, Germany) following the manufacturer’s instructions. Total RNA was extracted with a QIAamp® Viral RNA Kit (Qiagen, Germany) and was reverse-transcribed to cDNA with reverse transcriptase and dNTPs (Thermo Fisher, USA). Libraries were constructed for the DNA and cDNA samples with a Nextera XT DNA Library Prep Kit (Illumina, San Diego, CA) [9]. Library quality was assessed with a Qubit dsDNA HS Assay kit or High Sensitivity DNA kit (Agilent) on an Agilent 2100 Bioanalyzer. Library pools were then loaded onto an Illumina Nextseq CN500 sequencer for 75 cycles of paired-end sequencing to generate approximately 20 million reads for each library [9,13]. Trimmomatic was used to remove low-quality reads, adapter contamination, and duplicate reads, as well as those shorter than 50 bp [14]. Low-complexity reads were removed by Kcomplexity with default parameters [15]. Human sequence data were identified and excluded by mapping to a human reference genome (hg38) using Burrows–Wheeler Aligner software [16]. Full genome sequences of the influenza A virus from the patient were assembled by megaHIT v1.2.9 [17] and the pathogen complete genome was deposited in the Genome Warehouse in the National Genomics Data Center (National Genomics Data Center Members and Partners, 2021) under project PRJCA010960, which are publicly accessible at https://bigd.big.ac.cn/gsa. For the homologous and phylogenetic analysis, the resulting sequences were aligned using BLAST with the National Center for Biotechnology Information non-redundant nucleotide database [18]. All the sequences of eight segments of the avian influenza A virus from Genbank were downloaded. The genomic coverage graph was drawn by home-made scripts.

Tracheal aspirate specimens, CSF, and serum collected from the patient on day 6 after illness onset were centrifuged at 12000rpm for 10 min at 4°C to remove potential bacterial contamination, and then were inoculated into MDCK cells or 10-day-old SPF embryonated chicken eggs at 35°C, respectively. RNA from MDCK cell culture supernatant and chick embryo allantoic fluid was extracted with RNA isolator (Vazyme, China). Specific RT–PCR for influenza H1 to H15 subtypes was used to verify the viral subtype, as previously described [19].

RNA from the patient’s CSF was extracted with RNA isolator (Vazyme, China), according to the manufacturer’s instructions, and was reverse-transcribed to cDNA with HiScript II 1st Strand cDNA Synthesis Kit (Vazyme, China). The full genome of the virus was amplified with Phanta Max Super-Fidelity DNA Polymerase (Vazyme, China) and PCR products were purified from agarose gel with the Wizard® SV Gel and PCR Clean-Up System (Promega, USA) and were sent to Sangon Biotech (Shanghai, China) for sequencing. Full genome sequences of the virus were submitted to the Global Initiative on Sharing Avian Influenza Data database (accession number EPI2042509-16) and National Center for Biotechnology Information (accession number OP209766-73). A maximum likelihood phylogenetic tree based on the ORF of each segment of the selected influenza A viruses was constructed with MEGA7.0 and the highest identity to known strains was analyzed with DNAStar. The universal primer sets for full genome sequencing were described by Hoffumann et al [20].

## Results

### Case report

**Figure 1.**
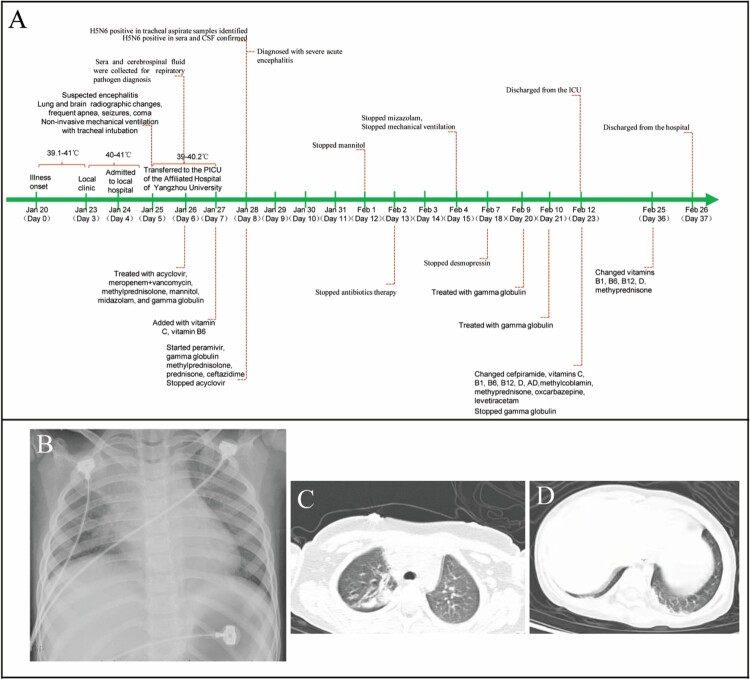
Timeline and chest imaging. Panel A is a timeline of the clinical course of the patient (PICU), paediatric intensive care unit; CSF, cerebrospinal fluid. Chest radiography (Day 6 of illness) indicated bilateral patchy shadows with consolidation in the right lung upper lobe (Panel B). Chest computed tomography showed consolidation of the right lung upper lobe (Panel C) and mild consolidation of the bilateral lower lobe near the pleura (Panel D).

**Figure 2.**
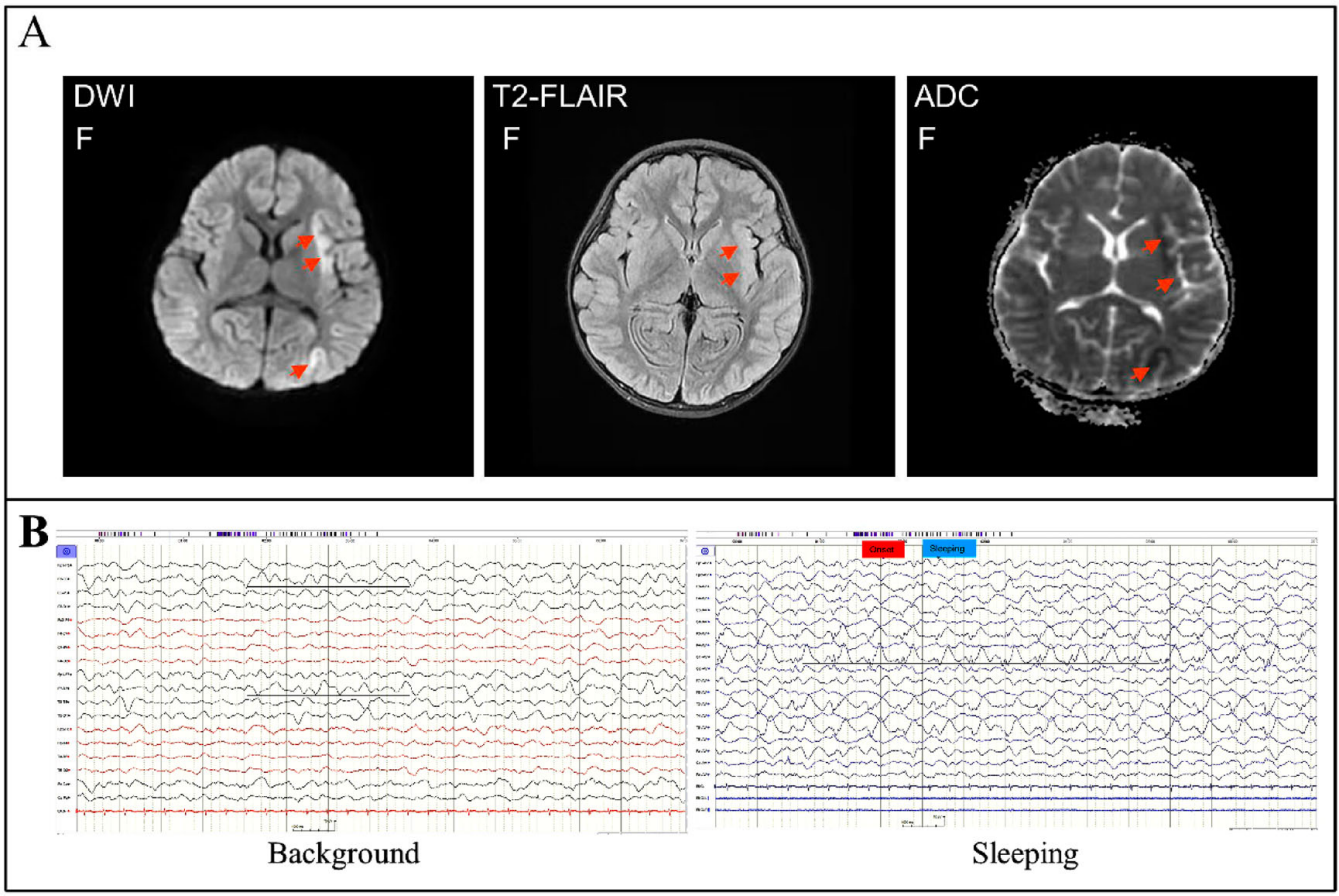
Brain imaging and Electroencephalogram (EEG). Panel A: Magnetic resonance imaging (MRI) of the brain performed on day 6 of the illness showed obvious abnormal signals on DWI (abnormally hyperintensity in the left insula and parietal lobe), ADC (abnormally hypointensity in the left insula and parietal lobe), and T2-FLAIR images (slightly hyperintensity in the left insula lobe) (marked with an arrow). Panel B: EEG on day 6 of the illness presented the relative enhanced θ and δ activities with the continuous asymmetrical low waves manifested spike, sharp, or slow wave complex (marked with a black line).

On January 20, 2022, fever, headache, dizziness, walking instability, and sleep increase were observed in a previously healthy 6-year-old girl with a maximal 39.1°C body temperature but no apparent respiratory symptoms. After treatment with antibiotics for one day, her symptoms have not improved, her fever persisted, and her body temperature fluctuated at 39.0-40.0°C. On Jan 21, 2022, she was admitted at a local hospital with a suspected bacterial infection and treated with azithromycin, penicillin, cefoperazone, or ceftazidime for 3 days. However, she still had a persistent fever with a body temperature of up to 41.0°C with no improvement at all, and she became lethargic. On Jan 25, 2022, she rapidly developed symptoms typical of acute encephalitis including lethargy, impassive and mask-like face, limb weakness, vertigo, tremours, aphasia, torticollis, opisthotonus, epileptic seizures, and convulsion. Therefore, she was urgently transferred to the paediatric intensive care unit (PICU) of the affiliated hospital of Yangzhou university (Figure 1A). On admission, she rapidly progressed to seizures and coma (Glasgow Coma Scale score, 7) and developed frequent apnoea and irregular breathing with low oxygen saturation, and was commenced non-invasive mechanical ventilation (Supplementary Tables). Physical examination revealed that positive for Brudzinski’s sign and bilateral Babinski’s sign, but negative for Kernig’s sign. Haematological examination revealed a significant increase in white cell (21.45 × 10^9^ cells/L), neutrophils (18.31 × 10^9^ cells/L), and lymphmonocytes (1.28 × 10^9^ cells/L) (Table 1). Blood biochemical parameters showed that markedly elevated alanine aminotransferase (68 U/L), aspartate aminotransferase (132 U/L), γ-glutamyl transpeptidase (143 U/L), lactate dehydrogenase (1041 U/L), creatine kinase (269 U/L), and creatine kinase isoenzyme (294.9 U/L) (Table 1). Chest radiograph indicated bilateral patchy shadows with consolidation in the right lung upper lobe (Figure 1B). Chest computed tomography showed consolidation of the right lung upper lobe and mild consolidation of the bilateral lower lobe (Figure 1C and D). MRI showed obvious abnormal signals in the right parietal lobe, bilateral frontal lobe, insula, and temporo-occipital lobe on the diffusion-weighted imaging (DWI), apparent diffusion coefficient (ADC) images, and T2 fluid-attenuated inversion-recovery (T2-FLAIR) images (Figure 2A). EEG presented the relative enhanced θ and δ activities (Figure 2B). A lumbar puncture was performed, and CSF on day 6 of the illness was examined. CSF contained 2 × 10^6^/L of red blood cells, 546 × 10^6^/L of white cells (mononuclear cells dominant), and 1.291 g/L of protein (Supplementary Tables). Cytokine examination for the patient’s serum revealed that the levels of interleukins (IL-1β, IL-2, IL-4, IL-5, IL-6, IL-10, and IL-12P70), interferon-α (IFN-α), tumour necrosis factor α (TNF-α) were significantly elevated (Table 1).

**Table 1.** Clinical blood routine and blood biochemical parameters.

Subjects	Reference	Sample collected time (Days of the illness)						
		5	6	7	10	12	16	20
WBC counts (x10^9^/L)	5.0–12.0	21.45	29.56	24.00	10.91	16.22	15.25	7.64
Neutrophils (x10^9^/L)	2–7	18.31	26.19	18.15	6.85	8.68	10.51	5.48
Ratio of neutrophils (%)	50–70	85.40	88.60	75.70	62.7	53.5	68.9	71.7
Lymphmonocytes (x10^9^/L)	0.12–0.8	1.28	2.36	2.33	1.01	1.45	1.03	0.43
Alanine aminotransferase (U/L)	9–52	68	86	55	36	36	38	45
Aspartate aminotransferase (U/L)	14–36	132	179	95	116	109	89	77
r-glutamyl transpeptidase (U/L)	12–43	143	169	136	90	84	65	55
Alkaline Phosphatase (U/L)	38–126	147	180	126	79	85	91	87
Lactate dehydrogenase (U/L)	101–240	1041	557	520	813	934	814	576
Creatine kinase (U/L)	30–135	269	75	49	34	59	72	/
Creatine kinase isoenzyme (U/L)	0–16	294.9	26	22	25	18	17	/
High-sensitivity c-reactive protein (mg/L)	0.1–8.2	71.0	90.08	43.78	5.66	1.73	0.31	0.37
IL-1β (pg/ml)	0–12.4	/	/	20.90	/	/	/	/
IL-2 (pg/ml)	0–5.71	/	/	7.57	/	/	/	/
IL-4 (pg/ml)	0–3.0	/	/	7.46	/	/	/	/
IL-5 (pg/ml)	0–3.1	/	/	6.47	/	/	/	/
IL-6 (pg/ml)	0–5.3	/	/	11.10	/	/	/	/
IL-10 (pg/ml)	0–4.91	/	/	11.65	/	/	/	/
IL-12P70 (pg/ml)	0–3.4	/	/	12.40	/	/	/	/
IFN-α (pg/ml)	0–8.5	/	/	29.36	/	/	/	/
TNF-α (pg/ml)	0–4.6	/	/	5.78	/	/	/	/

Note: “/”, no examination.

### Clinical treatment

**Figure 3.**
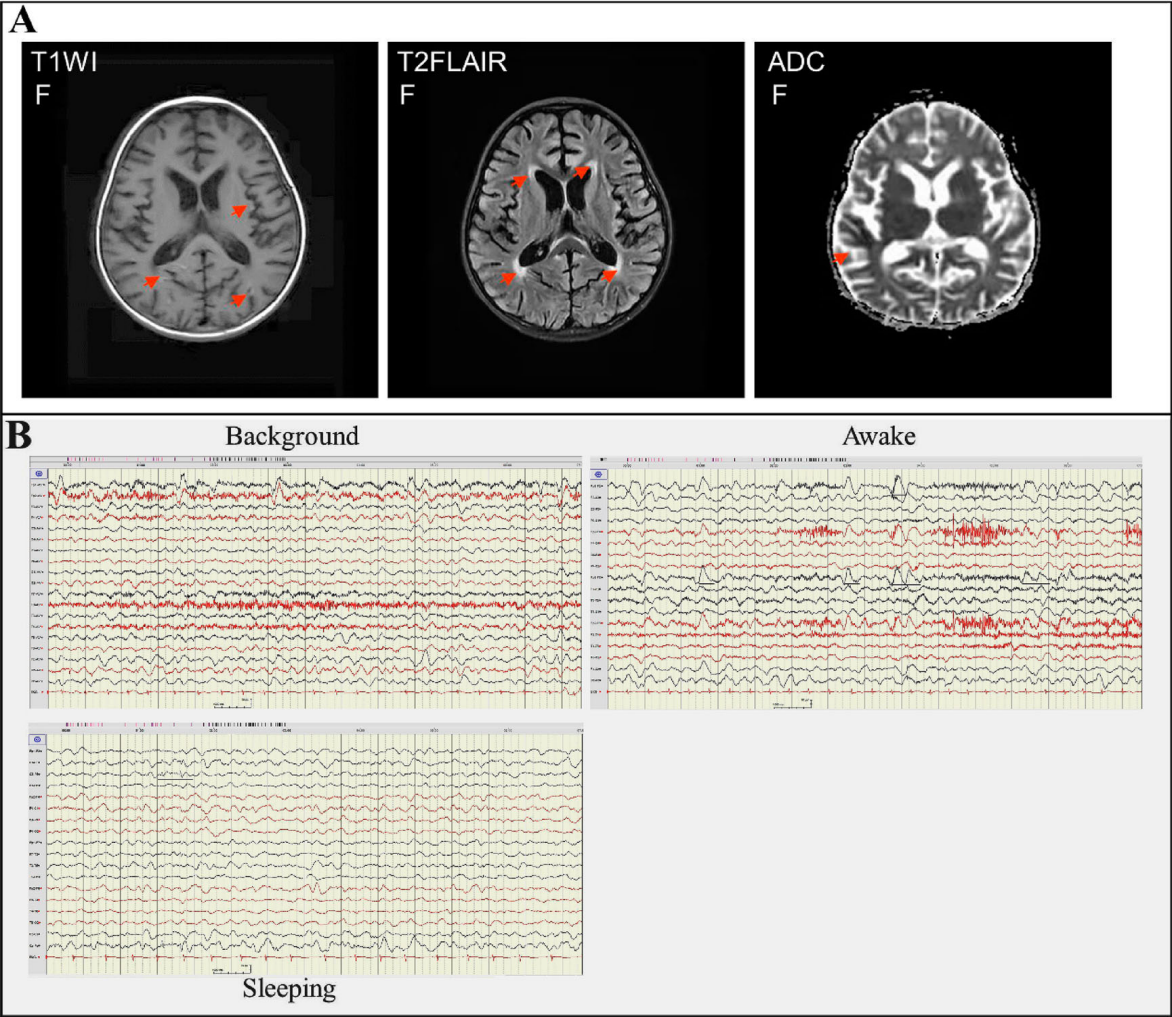
Brain imaging and EEG on day 28 of the illness. Panel A: MRI of the brain indicated abnormal signals on DWI, ADC, and T2-FLAIR images (marked with an arrow). DWI showed lamellar necrosis of the cerebral cortex; T2-FLAIR indicated cerebral sulci, bilateral lateral fissure cisterns, and lateral ventricle enlargement. Panel B: EEG presented the relative enhanced θ and δ activities with the continuous asymmetrical low waves manifested spike, sharp, or slow wave complex (black line marked).

Before the main causative pathogen was identified, the patient was treated empirically with broad-spectrum antibiotics for antimicrobial therapy due to elevated serum procalcitonin. The patient received intravenously 20 mg of meropenem per kilogram of body weight per 8 h (20 mg/kg/8 h) from day 5 to day 13 of illness onset and 10 mg/kg/6 h of vancomycin from day 6 to day 13 of illness onset and then changed to ceftazidime (100 mg/kg/d) treatment from day 13 to day 17 of illness onset. Due to suspected viral encephalitis, the patient was treated empirically with acyclovir (10 mg/kg/8 h) for antiviral therapy before the pathogen has been determined. After confirming that the pathogen is the H5N6 virus, the patient was changed to treat intravenously with 150 mg/day of peramivir for 3 days. To improve immunity, the patient received intravenously 2 times of human gamma globulin (2 g/kg) treatment on day 5 to day 7, and day 9 to day 10 of the illness. In addition, the patient received intravenously 20 mg of methylprednisolone per 12 h for 4 days, 340 mg of methylprednisolone per day for 3 days, and 17 mg of methylprednisolone per 2 h for 7 days. To reduce the patient’s intracranial pressure, the patient was treated with 25% mannitol for 8 days from day 5 to day 13 of the illness. Due to the frequent occurrence of convulsions and epilepsy, the patient was treated with midazolam from day 4 to day 15 of the illness, and oxcarbazepine and levetiracetam were added from day 5 to day 15 of the illness. Mechanical ventilation was removed on day 15 of the illness and nasogastric feeding was stopped on day 30. Brain MRI on day 28 of illness still showed abnormal signals including gyri-like hyperintensities in the bilateral frontotemporal parietal occipital lobes on the diffusion-weighted imaging (T1WI), slightly hyperintensities in the bilateral frontotemporal parietal occipital lobes and patchy hyperintensities in the anterior and posterior horns of bilateral ventricles on the T2 fluid-attenuated inversion-recovery (T2-FLAIR) images, patchy and gyri-like hypointense signals in the right parietal and temporal lobes on the apparent diffusion coefficient (ADC) images (Figure 3), indicating that the brain injury has not fully recovered. EEG on day 31 of illness still presented the relative enhanced θ and δ activities on the left side, including a slow background, continuous left-right asymmetry, scattered multifocal slow wave discharges on the left side during the stage of waking and sleeping, and a small number of slow waves as a background activity with spike, sharp, or slow wave complex on the left central, top, and temporal regions at the stage of the sleeping period (Figure 3), indicating abnormal brain waves. The girl was discharged from the hospital on day 32 of illness onset (26 February). Except for that language expression, memory, voluntary urination and defaecation have not yet fully recovered, no other apparent neurological or developmental delay was observed during follow-up two to four months after discharge. But 6 months later she shows obvious features of brain atrophy.

### Pathogen investigation

**Figure 4.**
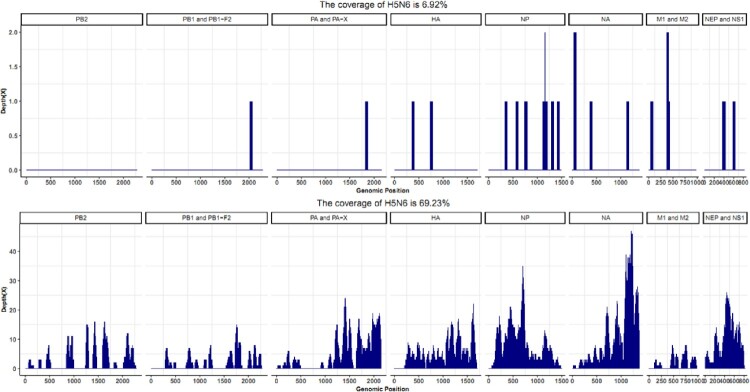
mNGS results of the patient’s CSF and serum revealed that the H5N6 virus was the overwhelmingly microbial species. Panel A: mNGS data of viral RNA from the patient’s serum. Panel B: mNGS data of viral RNA from the patient’s CSF.

No pathogenic bacteria or fungi were isolated from the blood, urine, and CSF. mNGS for the patient’s serum and CSF indicated no bacterial, fungal, or parasitic infections. SARS-CoV-2, HBV antigen, HCV antigen, HIV antigen, HIV antibodies, influenza A virus H1 and H3 subtype antibodies IgM, influenza B virus antibodies IgM, Adenovirus (ADV) antibodies IgM, RSV antibodies IgM, and chlamydia pneumonia antibodies IgM were negative. Mycoplasma pneumonia antibodies IgM was negative, but MP antibodies IgG was 172.00 AU/ml, much higher than the normal range. Human parainfluenza virus (HPIV) antibodies IgM was positive, but HPIV antigen was negative. EBV antibodies IgM and IgA were negative, but EBV antibodies IgG was positive. The result of RT–PCR from the patient’s serum, CSF, and throat specimens indicated H5N6 positive, but negative for twenty-one other respiratory pathogens including seasonal influenza A viruses (H1, H3, or B). The virus was also isolated from the tracheal aspirate specimens collected on day 7 of illness onset. The mNGS data from the CSF and serum collected on day 7 revealed that avian influenza A H5N6 virus was the overwhelmingly microbial species (Figure 4). Virus isolation from the CSF and serum also confirmed the above results. The CSF and serum contained infectious virus titres of 10^2.5^ TCID_50_/50 µl and 10^1.0^TCID_50_/50 µl, respectively. A novel reassortant H5N6 was designated as A/Yangzhou/125/2022 (YZ125) isolated from the patient’s CSF and was used for further analysis. The haemagglutination inhibition (HI) antibody titres of the serum and CSF against the YZ125 virus was 256 and 4, respectively. All evidence indicated that the H5N6 virus was considered the most likely pathogen.

### Phylogenic and mutation analysis

**Figure 5.**
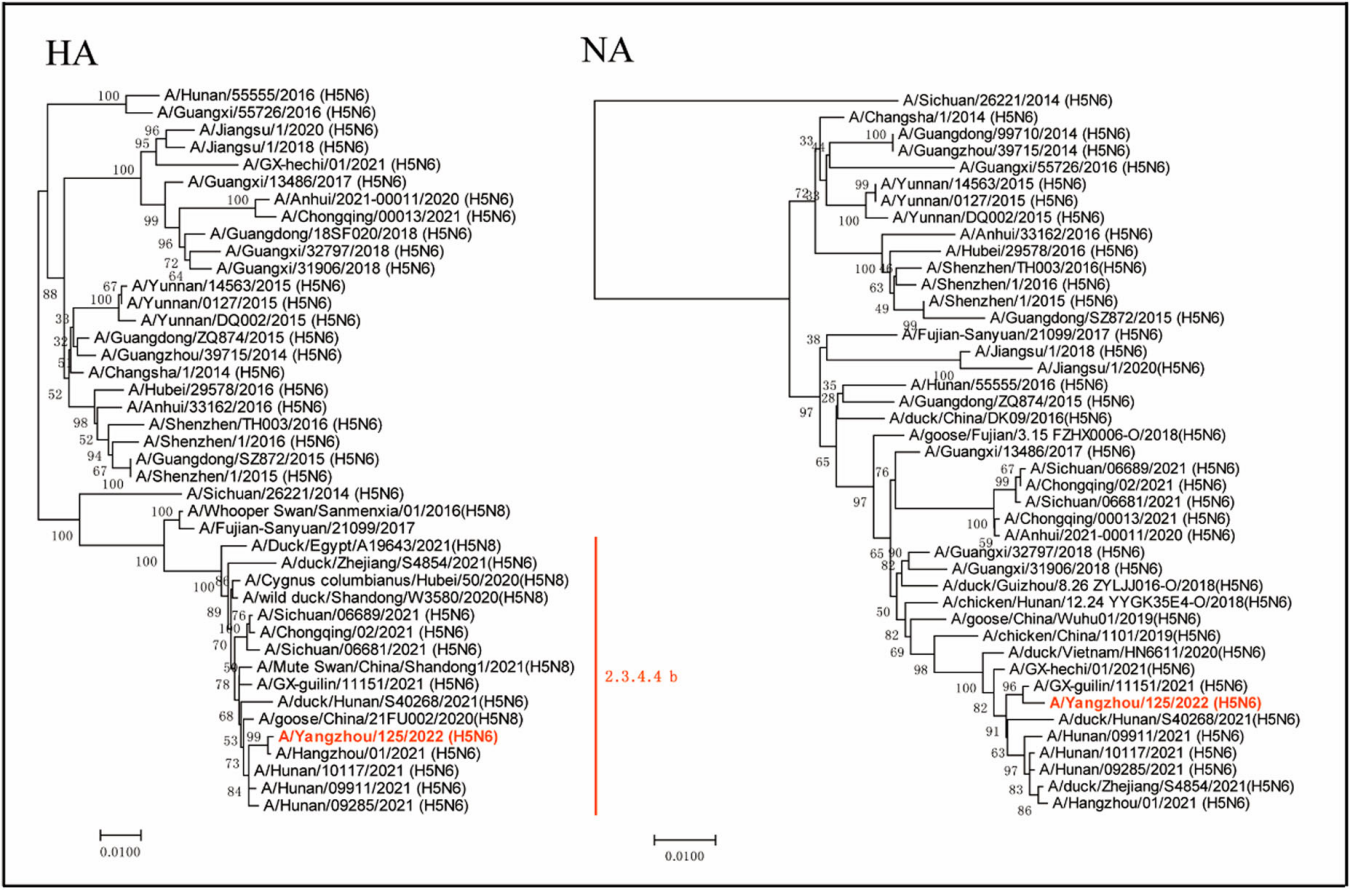
Phylogenetic trees of haemagglutinin (HA) and neuraminidase (NA) genes. The novel H5N6 isolate (YZ125) was marked with red. Clade lineages of human-infected avian influenza viruses are indicated on the right panel. Phylogenetic trees of each segment were constructed with MEGA7.0 software (https://www.megasoftware.net) using the neighbour-joining method and the sequence of ORF of each gene segment. Bootstrap analysis was performed with 1,000 replications. Scale bars indicated nucleotide substitutions per site.

Phylogenic analysis indicated that the YZ125 virus was a novel reassortant H5N6 virus that belonged to clade 2.3.4.4b (Figure 5 and Supplementary Figures). Although no known H5N6 genomes completely matched our sequences, the eight genes of YZ125 shared the highest identity (99.3–100%) with A/Hangzhou/1/2021 (H5N6) or A/duck/Zhejiang/S4854/2021 (H5N6) (Supplementary Tables). The HA cleavage site of the YZ125 virus contains a multiple basic amino acids motif of HPAIV (PLREKRRKR/G). The amino acid motif at residues 226–228 (H3 numbering) in HA was Gln-Ser-Gly, suggesting its preference for an avian-like receptor [21]. However, the receptor-binding site Thr160Ala mutation might enhance affinity with the human-type receptor. A deletion of 12 amino acids at positions 59–70 in the NA stalk and 5 amino acids at positions 80–84 in the NS1 associated with increased virulence in mice were observed [22]. Furthermore, many mutations in the internal genes associated with enhanced virulence in mice, including Leu89Val, Gly309Asp, Thr339Lys, Arg477Gly, Ile495 Val, Ala676Thr in PB2 [23], Ser622Gly in PB1[24], Ser515Thr in PA [25], Asn30Asp, Thr215Ala, Ile43Met in M1 [26,27], Pro42Ser [28], Val149Ala [29], Cys138Leu [30], Leu103Phe [31], Ile106Met [32] in NS1 were revealed (Supplementary Tables). No mutations associated with drug resistance were found in the M2 and NA proteins. *In vitro* antiviral tests from the MDCK cell infection model also confirmed that the EC_50_ of oseltamivir and peramivir on the YZ125 virus was approximately 0.14 µg/ml and 1.4 ng/ml, respectively, suggesting that the YZ125 virus remains sensitive to neuraminidase inhibitors.

### Epidemiological investigation

After the diagnosis has been done, the patient’s village and house were surveyed. Her family and neighbours were interviewed. The family lives in a small village next to the Baita River nearby the Yangtze River. There is a large inner lake connected to the Baita River in front of her house. Many wild waterfowl and domestic ducks are living together in the lake. Before the illness onset, she often liked to go to the lake for watching wild waterfowl and domestic ducks. Even in her house, wild bird droppings can be seen everywhere. Moreover, during the illness onset, her pet dog died and she had close contact with the dog, but no epidemiological diagnosis for the pet dog was done. Her family has no poultry and did not handle poultry from markets. No signs of influenza-like symptoms in all close contacts were observed during her illness. However, due to the slaughter of domestic ducks and the burial of pet dogs, no direct evidence of H5N6 virus infection was obtained from the above samples. Notably, 4 months after the occurrence of the case, one HPAI (H5N6) virus highly homologous to the YZ125 (H5N6) virus was isolated from the faeces of local wild waterfowls where the case occurred, suggesting that wild waterfowls may be the direct source of this infection.

## Discussion

Influenza A virus is primarily limited to the respiratory system, but many of the reported cases of influenza A viruses, including the “1918 H1N1” influenza pandemic, had neurological symptoms typical of encephalitis, which can result in severe neurological sequelae and even death [33,34]. Influenza-associated encephalitis and encephalopathy (IAE) caused by influenza A H3N2 or H1N1 are most frequently reported in children although adult cases are described [34,35]. However, acute neurological symptoms from mild encephalitis to motor disturbances to coma caused by HPAIV have been observed mainly in poultry and wild mammals [36,37]. In addition, most patients infected by HPAIV, including H5N1, H5N6, and H7N9, exhibit primarily acute respiratory illness, and rarely neurological symptoms have been reported [1–3]. To date, there are only 3 case reports of H5N1 infections with encephalitis, but only one fatal case was confirmed by isolating the H5N1 virus from the stored CSF [38,39]. However, due to no increase in white cells in CSF and the lack of brain imaging, it cannot conclusively determine whether the patient had true encephalitis [39]. Virological diagnosis is a prerequisite for determining whether AIE occurs. To the best of our knowledge, most reports of IAE were mainly based on the detection of influenza A virus RNA from CSF with rare evidence of isolating influenza A virus [33,34]. In addition, excessive cytokine storm was observed in most patients with IAE [33]. Therefore, the current prevailing theory is that IAE might be associated with hypercytokinaemia rather than actual viral invasion. However, the pathogenic mechanisms of IAE remain largely unknown. In this study, our results clearly indicated that the patient’s acute encephalitis was caused by an H5N6 virus infection. The diagnosis of acute encephalitis was based on the following evidence: neurological symptoms, brain imaging, EEG, biochemical characteristics of CSF and serum specimens, serology analysis, and virus isolation. The virological diagnosis was confirmed by RT–PCR, mNGS, and virus isolation from serum, CSF, and throat-swab specimens. Due to the identification of pathogens in all clinical specimens performed by different staff at separate places on different dates apart (Yangzhou Center for Disease Control and Prevention, Vision Medicals Co. Ltd (Guangzhou, China), and Yangzhou University), the possibility of laboratory contamination can be completely ruled out. On-going *in vivo* work will reveal the potential pathogenesis of viral encephalitis caused by this virus.

In previously reported cases of IAE, clinical symptoms of influenza include but are not limited to fever, headache, pharyngitis, and myalgia [33,34,40].This particular patient had symptoms of fever, headache, dizziness, vomiting, walking instability, language reduction, and sleep increase with elevated white cells and neutrophils, but had no obvious respiratory symptoms on illness onset. Therefore, it may be suspected as bacterial infection on illness onset. However, after 5 days of antibiotic treatment, not only did her symptoms not improve significantly but instead rapidly progressed to typical symptoms of encephalitis, such as convulsions, epilepsy, and coma. Therefore, acute encephalitis was suspected, and antiviral therapy was given empirically with acyclovir. After the diagnosis of H5N6 virus infection, the patient was changed to peramivir treatment. In the previous literature, steroid pulse therapy in the acute phase of IAE has beneficial effects on patient morbidity and mortality [41,42]. In view of excessive cytokines storms in sera, she received steroid pulse therapy with methylprednisolone. In addition, our experience suggests that the specialized supportive therapies of PICU including mechanical ventilation, early convulsion control, reduction of cranial pressure and oedema, anti-epileptic treatment, fluid management, and nutritional support may play an important role in the improvement of the prognosis of this case. Therefore, our research will provide a reference for clinicians, virologists, and public health experts to guide clinical treatment, laboratory diagnosis, and epidemiological surveillance for this potential pandemic disease.

Due to the patient’s family neither raised poultry nor slaughtered poultry, the possibility of exposure to ill poultry should be ruled out. Moreover, long-term epidemiologic surveillance from our lab indicated that there was no this novel H5N6 reassortant circulating in poultry in Yangzhou city. Additionally, there were large amounts of wild waterfowl and domestic ducks living together in the inner lake in front of the patient’s house, we speculate that the most likely source of transmission might be wild waterfowl overwintering in the lake. The above speculation is further supported by the latest epidemiological reports, suggesting that these novel H5N6 reassortants including A/Hangzhou/1/2021 and A/duck/Zhejiang/S4854/2021, which are most closely related to the YZ125 virus, may be generated through reassortment between migratory wild birds and domestic ducks. Recently, we isolated one H5N6 virus shared with highly homologous to the YZ125 (H5N6) virus from the faeces of wild waterfowl where the patient lives, further confirming the above speculation. Moreover, it is worth noting that this YZ125-like virus has successively infected humans in Guangxi, Hunan, Zhejiang, and Jiangsu provinces since 2021[5–7,43], suggesting that these novel reassortants H5N6 viruses might get the ability to directly infect human without adaption. Because the clinical manifestations of this novel H5N6 reassortant are acute encephalitis, rather than previous respiratory symptoms, once these reassortants obtained the ability of human-to-human transmission through reassortment or mutations, it will bring great health threat to humans. Therefore, it is a very urgent need to enhance the epidemiological surveillance of wild waterfowl and to take effective measures to prevent close contact between wild waterfowl and poultry in areas where migratory birds frequently migrate.

## Supplementary Material

Supplemental Material
