# Supplementary material for: Clinical features of the first critical case of acute encephalitis caused by the avian influenza A (H5N6) virus
Source: Emerg Microbes Infect. 2022 Oct 26;11(1):2437–46. doi: 10.1080/22221751.2022.2122584 (PMC9621215; doi:10.1080/22221751.2022.2122584)
Supplement: Supplemental Material [file TEMI_A_2122584_SM6061.zip › Supplementary figures.docx]

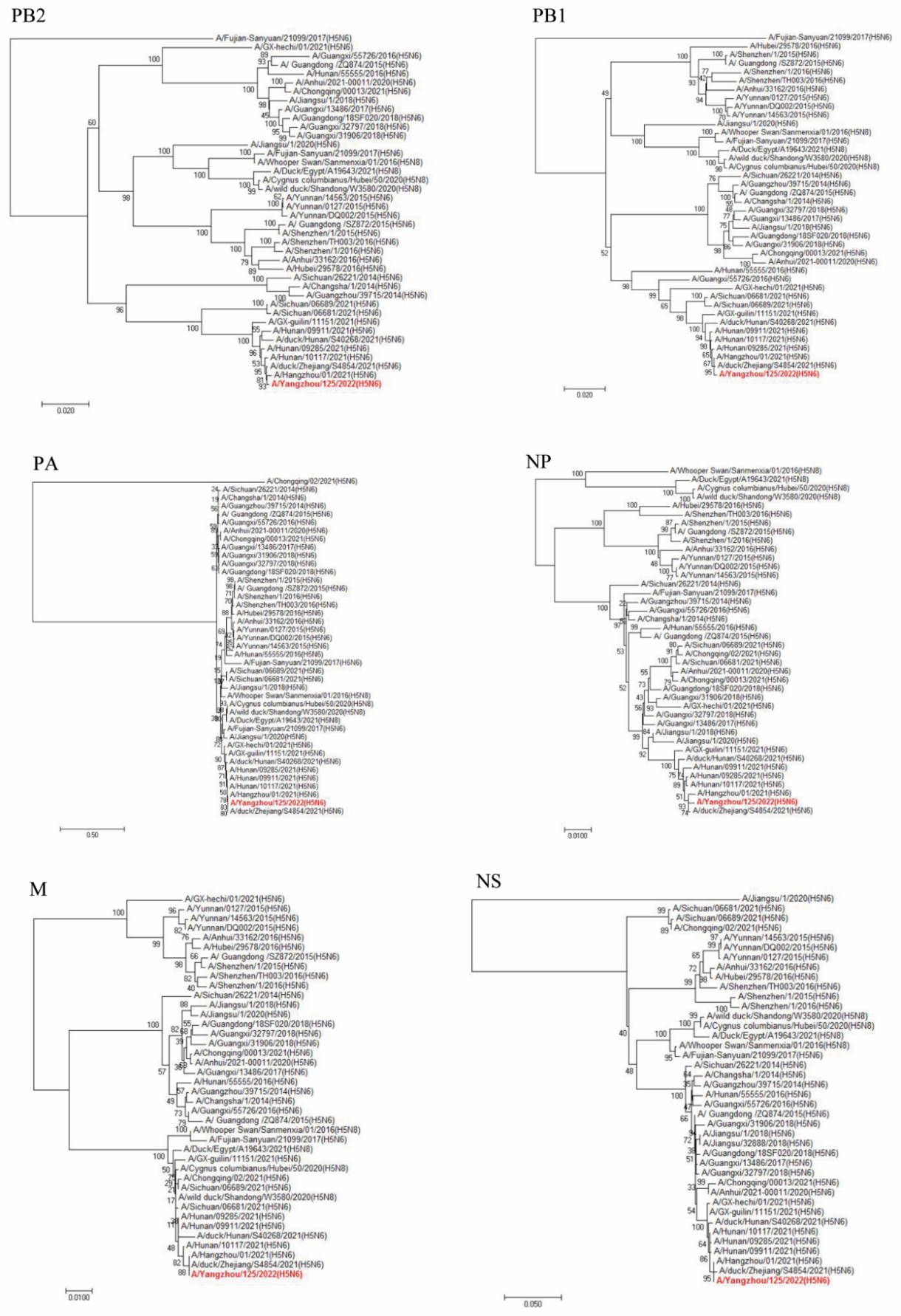


Supplementary figure. Phylogenetic trees of PB2, PB1, PA, M and NS genes. The novel H5N6 isolate (YZ125) was marked with red. Phylogenetic trees were constructed with MEGA7.0 software (https://www.megasoftware.net) using the neighbor-joining method and the sequence of ORF of each gene. Bootstrap analysis was performed with 1,000 replications. Scale bars indicated nucleotide substitutions per site.
