# Supplementary material for: Clinical features of the first critical case of acute encephalitis caused by the avian influenza A (H5N6) virus
Source: Emerg Microbes Infect. 2022 Oct 26;11(1):2437–46. doi: 10.1080/22221751.2022.2122584 (PMC9621215; doi:10.1080/22221751.2022.2122584)
Supplement: Supplemental Material [file TEMI_A_2122584_SM6061.zip › Supplementray Tables.docx]

| Subjects | Reference | Data collected time (Days of illness) | | | | | | | |
| --- | --- | --- | --- | --- | --- | --- | --- | --- | --- |
|  |  | 5 | 6 | 7 | 8 | 9 | 10 | 11 | 12 |
| PH | 7.35-7.45 | 7.22 | 7.47 | 7.48 | 7.35 | 7.56 | 7.50 | 7.50 | 7.48 |
| PCO2(mmHg) | 35-48 | 73 | 39 | 41 | 32 | 30 | 29 | 36 | 35 |
| PO2(mmHg) | 83-108 | 53 | 137 | 152 | 131 | 126 | 121 | 108 | 122 |
| LAC(mmol/L) | 0.5-2.2 | 1 | 0.80 | 0.90 | 1.50 | 1.30 | 1.20 | 1.70 | 1.20 |
| HCO3(mmol/L) | 18-23 | 29.9 | 28.4 | 30.5 | 26.7 | 26.9 | 22.6 | 28.1 | 26.1 |
| HCO3-std(mmol/L) | 18-23 | 25.2 | 28.4 | 30.0 | 28.0 | 28.6 | 24.8 | 28.6 | 26.9 |
| TCO2(mmol/L) | 22-29 | 32.1 | 29.6 | 31.8 | 27.7 | 27.8 | 23.5 | 29.2 | 27.2 |
| blood oxygen saturation (%) | 95-98 | 79 | 99 | 99 | 99 | 99 | 99 | 99 | 99 |

**Suppl. Table 1. Clinical blood gas analysis parameters**

| Blood parameters | Reference | Sample collected time (Days of illness) | | | | | | | |
| --- | --- | --- | --- | --- | --- | --- | --- | --- | --- |
|  |  | 5 | 6 | 7 | 10 | 12 | 16 | 19 | 26 |
| WBC counts (x10^9^/L) | 5.0-12.0 | 21.45 | 29.56 | 24.00 | 10.91 | 16.22 | 15.25 | 7.64 | 8.61 |
| Neutrophils (x10^9^/L) | 2-7 | 18.31 | 26.19 | 18.15 | 6.85 | 8.68 | 10.51 | 5.48 | 5.49 |
| Ratio of neutrophils (%) | 50-70 | 85.40 | 88.60 | 75.70 | 62.7 | 53.5 | 68.9 | 71.7 | 63.9 |
| Lymphocytes (x10^9^/L) | 0.8-4 | 1.80 | 0.95 | 3.52 | 3.04 | 6.05 | 3.70 | 1.73 | 2.56 |
| Ratio of lymphocytes (%) | 20-40 | 8.40 | 3.20 | 14.60 | 27.9 | 37.3 | 24.3 | 22.6 | 29.7 |
| Lymphmonocytes (x10^9^/L) | 0.12-0.8 | 1.28 | 2.36 | 2.33 | 1.01 | 1.45 | 1.03 | 0.43 | 0.52 |
| Ratio of lymphmonocytes (%) | 3-8 | 6.0 | 8.0 | 9.70 | 9.3 | 8.9 | 6.8 | 5.6 | 6.0 |
| Eosinophils (x10^9^/L) | 0.05-0.5 | 0.05 | 0.06 | 0.00 | 0.00 | 0.01 | 0.01 | 0.00 | 0.02 |
| Ratio of eosinophils (%) | 0.5-5 | 0.20 | 0.20 | 0.00 | 0.0 | 0.1 | 0.0 | 0.0 | 0.2 |
| Basophils (x10^9^/L) | 0-0.1 | 0.01 | 0.00 | 0.00 | 0.01 | 0.03 | 0.00 | 0.00 | 0.02 |
| Ratio of basophils (%) | 0-1 | 0.00 | 0.00 | 0.00 | 0.1 | 0.2 | 0.0 | 0.1 | 0.2 |
| RBC counts (x10^9^/L) | 3.8-5.1 | 5.07 | 3.99 | 3.63 | 3.19 | 3.39 | 3.06 | 3.24 | 4.46 |
| Hemoglobin (g/L) | 120-140 | 131.0 | 112.0 | 101.0 | 85.0 | 92.0 | 89.0 | 94.0 | 126.0 |
| Platelet counts (x10^9^/L) | 125-350 | 186.0 | 263.0 | 427.0 | 475 | 670 | 597 | 472 | 492 |
| Platelet hematocrit (%) | 0.2-0.5 | 12.6 | 0.23 | 0.37 | 0.45 | 0.59 | 0.42 | 0.34 | 0.39 |
| Mean platelet volume (fL) | 9-13 | 20.3 | 8.80 | 8.60 | 9.40 | 8.70 | 7.10 | 7.30 | 7.80 |
| Mean corpuscular volume MCV (fL) | 82-100 | 79.1 | 82.3 | 84.7 | 81.80 | 81.10 | 84.80 | 88.20 | 86.30 |
| Mean corpsular hemoglobin MCH (pg) | 27-34 | 25.8 | 28.0 | 27.9 | 26.60 | 27.10 | 29.00 | 29.0 | 28.30 |
| Mean corpsular hemoglobin concentration (g/L) | 316-354 | 327.0 | 340.0 | 329.0 | 326.0 | 335.0 | 342.0 | 329.0 | 327.0 |
| Red blood cell distribution width (%) | 11-16 | 13.3 | 12.1 | 12.7 | 11.40 | 11.30 | 12.50 | 15.30 | 14.40 |
| Hematocrit (%) | 33-42 | 40.1 | 32.8 | 30.7 | 26.10 | 27.50 | 26.00 | 28.60 | 38.50 |
| Platelet distribution width DW (fL) | 9-17 | 0.23 | 15.90 | 15.60 | 9.20 | 8.30 | 15.10 | 15.10 | 7.60 |
| High-sensitivity c-reactive protein (mg/L) | 0.1-8.2 | 71.0 | 90.08 | 43.78 | 5.66 | 1.73 | 0.31 | 0.37 | <0.50 |

**Suppl.Table 2. Clinical blood parameters**

| Subjects | Reference | Sample collected time (Days of illness) | | | | | | |
| --- | --- | --- | --- | --- | --- | --- | --- | --- |
|  |  | 5 | 6 | 7 | 10 | 12 | 16 | 20 |
| Total protein (g/L) | 63-82 | 71.3 | 58.5 | 65.7 | 77.6 | 80.1 | 75 | 74.6 |
| Globin (g/L) | 35-50 | 37.5 | 33.4 | 29.3 | 36.8 | 40 | 40 | 40.5 |
| Albumin (g/L) | 18-32 | 33.8 | 25.1 | 36.4 | 40.8 | 40.1 | 35 | 34.1 |
| Ratio of albumin to globulin | 1.5-2.5 | 1.1 | 1.33 | 0.8 | 0.9 | 1 | 1.14 | 1.19 |
| Total bilirubin (µmol/L) | 0-24.0 | 16.87 | 0.8 | 2.6 | 5.3 | 3.8 | 2.6 | 0.8 |
| Conjugated bilirubin (µmol/L) | 0-5 | 3.53 | 0.1 | 0.1 | 0.1 | 0.1 | 0.1 | 0.1 |
| unconjugated bilirubin (µmol/L) | 0-19 | 13.3 | 0.7 | 2.5 | 5.2 | 3.7 | 2.5 | 0.7 |
| Alanine aminotransferase (U/L) | 9-52 | 68 | 86 | 55 | 36 | 36 | 38 | 45 |
| Aspartate aminotransferase (U/L) | 14-36 | 132 | 179 | 95 | 116 | 109 | 89 | 77 |
| r-glutamyl transpeptidase (U/L) | 12-43 | 143 | 169 | 136 | 90 | 84 | 65 | 55 |
| Alkaline Phosphatase (U/L) | 38-126 | 147 | 180 | 126 | 79 | 85 | 91 | 87 |
| Lactate dehydrogenase (U/L) | 101-240 | 1041 | 557 | 520 | 813 | 934 | 814 | 576 |
| Cholinesterase (U/L) | 4650-10440 | / | 5310.3 | 4438.8 | 4625.9 | 5470 | 5872.5 | 6209.8 |
| Glucose (mmol/L) | 4.1-5.9 | / | 8.04 | 7.37 | 4.99 | 5.39 | 5.85 | / |
| Urea (mmol/L) | 2.5-6.1 | 3.51 | 1.96 | 2.99 | 3.42 | 3.01 | 3.65 | / |
| Creatinine (µmol/L) | 46-92 | 25.3 | 31.8 | 25.9 | 18.4 | 18.2 | 17.4 | / |
| Uric acid (µmol/L) | 155-428 | 208 | 188.1 | 126.5 | 40.1 | 42.9 | 86.6 | / |
| Potassium (mmol/L) | 3.5-5.1 | / | 3.79 | 3.64 | 4.5 | 4.29 | 3.7 | / |
| Sodium (mmol/L) | 137-145 | / | 129.8 | 131.3 | 133.5 | 137.7 | 136.7 | / |
| Chlorine (mmol/L) | 98-107 | / | 96.8 | 95.6 | 99.8 | 99.9 | 100.6 | / |
| Calcium (mmol/L) | 2.1-2.55 | 3.51 | 2.11 | 1.95 | 2.3 | 2.33 | 2.36 | / |
| magnesium(mmol/L) | 0.70-1.00 | 0.98 | 0.78 | 0.8 | 0.98 | 0.89 | 0.81 | / |
| Inorganic Phosphate (mmol/L) | 0.81-1.45 | 1.52 | 1.27 | 0.92 | 1.22 | 1.09 | 1.37 | / |
| Creatine kinase (U/L) | 30-135 | 269 | 75 | 49 | 34 | 59 | 72 | / |
| Creatine kinase isoenzyme (U/L) | 0-16 | 294.9 | 26 | 22 | 25 | 18 | 17 | / |
| High-sensitivity troponin (pg/ml) | <17.5 | / | 41.8 | 18.6 | 4.8 | 1.8 | 1.1 | / |

**Suppl. Table 3. Clinical blood biochemistry parameters**

Note: “/”, no examination.

| Cerebrospinal fluid test | Reference | Sample collected time (Day of illness onset | | |
| --- | --- | --- | --- | --- |
|  |  | 6 | 8 | 17 |
| Color | Colorless | Colorless | Colorless | Colorless |
| Turbidity | Clear | Clear | Clear | Clear |
| Clot | No | No | No | No |
| Pandy's test | Positive | Positive | Positive | Positive |
| White blood cells (x10^6^/L) | 0-5 | 546 | 162 | 9 |
| Mononuclear cells ratio (%) | <30% | 0.7 | 0.84 | 0.78 |
| Multinuclear cells ratio (%) | <6% | 0.3 | 0.16 | 0.22 |
| Red blood cells (x10^6^/L) | 0 | 2 | 14 | 1 |
| Glucose (mmol/L) | 2.2-3.9 | 3.28 | 2.81 | 3.45 |
| Chloride (mmol/L) | 109-129 | 112 | 115.5 | 115.2 |
| Protein concentration (mg/L) | 120-600 | 1291 | 1646 | 1071 |

**Suppl. Table 4. Clinical parameters of cerebrospinal fluid**

**Table 5. Molecular analysis of the nucleotide identity of the A/Yangzhou/125/2022 (H5N6) virus.**

| Gene | Viruses with greatest nucleotide identity | Accession number | identity (%) |
| --- | --- | --- | --- |
| PB2 | A/duck/Zhejiang/S4854/2021 (H5N6) | EPI1997223 | 99.80 |
|  | A/Hangzhou/1/2021 (H5N6) | EPI1946727 | 99.90 |
| PB1 | A/duck/Zhejiang/S4854/2021 (H5N6) | EPI1997224 | 99.80 |
|  | A/Hangzhou/1/2021 (H5N6) | EPI1946728 | 99.90 |
| PA | A/duck/Zhejiang/S4854/2021 (H5N6) | EPI1997222 | 99.60 |
|  | A/Hangzhou/1/2021 (H5N6) | EPI1946729 | 99.30 |
| HA | A/Cygnus columbianus/Hubei/53/2020(H5N8) | MW505399.1 | 99.19 |
|  | A/Hangzhou/1/2021 (H5N6) | EPI1946730 | 99.80 |
| NP | A/duck/Zhejiang/S4854/2021 (H5N6) | EPI1997219 | 99.70 |
|  | A/Hangzhou/1/2021 (H5N6) | EPI1946731 | 99.50 |
| NA | A/duck/Zhejiang/S4854/2021 (H5N6) | EPI1997225 | 99.8 |
|  | A/GX-guilin/11151/2021 (H5N6) | EPI1887791 | 99.6 |
| MP | A/duck/Zhejiang/S4854/2021 (H5N6) | EPI1997221 | 100 |
|  | A/Hangzhou/1/2021 (H5N6) | EPI1946733 | 100 |
| NS | A/duck/Zhejiang/S4854/2021 (H5N6) | MW872826.1 | 99.80 |
|  | A/Hangzhou/1/2021 (H5N6) | EPI1946734 | 100 |

Note: The greatest nucleotide identity to A/Yangzhou/125/2022 (H5N6) was shown in the table.

Suppl.Table 6 Key mutations in the eight gene of A/Yangzhou/125/2022 (H5N6)

| Protein | Amino acid^†^/motif | Phenotype | References |
| --- | --- | --- | --- |
| HA^*^ | Cleavage site  (PLREKRRKR) | Polybasic cleavage motif of high pathogenicity avian influenza A virus | 10 |
|  | Thr160Ala | Enhanced binding to α-2,6-sialic acid receptor and transmission between guinea pigs | 11,12 |
| PB2 | Leu89Val, Gly309Asp, Thr339Lys, Arg477Gly, Ile495Val, Ala676Thr | Enhanced virulence and replication in mammals | 13 |
| PB1 | Ser622Gly | Enhanced polymerase activity and virulence in mice | 14 |
| PA | Ser515Thr | Enhanced polymerase activity in mammalian cells | 15 |
| M1 | Asn30Asp | Enhanced virulence in mice | 16 |
|  | Thr215Ala | Enhanced virulence in mice | 16 |
|  | Ile43Met | Enhanced virulence in mice, chickens and ducks | 17 |
| NS1 | Pro42Ser | Enhanced virulence in mice | 18 |
|  | Val149Ala | Enhanced virulence and attenuated interferon response in chickens | 19 |
|  | Cys138Leu | Enhanced replication in mammalian cells, attenuated interferon response | 20 |
|  | Leu103Phe, Ile106Met | Enhanced virulence in mice | 21,22 |

^*^The numbering of HA is relative to A/New York/392/2004(H3N2).

^†^The numbering of each amino acid /motif is relative to A/goose/Guangdong/1/1996(H5N1).
